# The dynamics of mitochondrial autophagy at the initiation stage

**DOI:** 10.1042/BST20210272

**Published:** 2021-10-19

**Authors:** Nicholas T. Ktistakis

**Affiliations:** Signalling Programme, Babraham Institute, Cambridge CB22 3AT, U.K.

**Keywords:** autophagy, endoplasmic reticulum, imaging techniques, mitochondria

## Abstract

The pathway of mitochondrial-specific autophagy (mitophagy, defined here as the specific elimination of mitochondria following distinct mitochondrial injuries or developmental/metabolic alterations) is important in health and disease. This review will be focussed on the earliest steps of the pathway concerning the mechanisms and requirements for initiating autophagosome formation on a mitochondrial target. More specifically, and in view of the fact that we understand the basic mechanism of non-selective autophagy and are beginning to reshape this knowledge towards the pathways of selective autophagy, two aspects of mitophagy will be covered: (i) How does a machinery normally working in association with the endoplasmic reticulum (ER) to make an autophagosome can also do so at a site distinct from the ER such as on the surface of the targeted cargo? and (ii) how does the machinery deal with cargo of multiple sizes?

## Introduction

**Figure 1.**
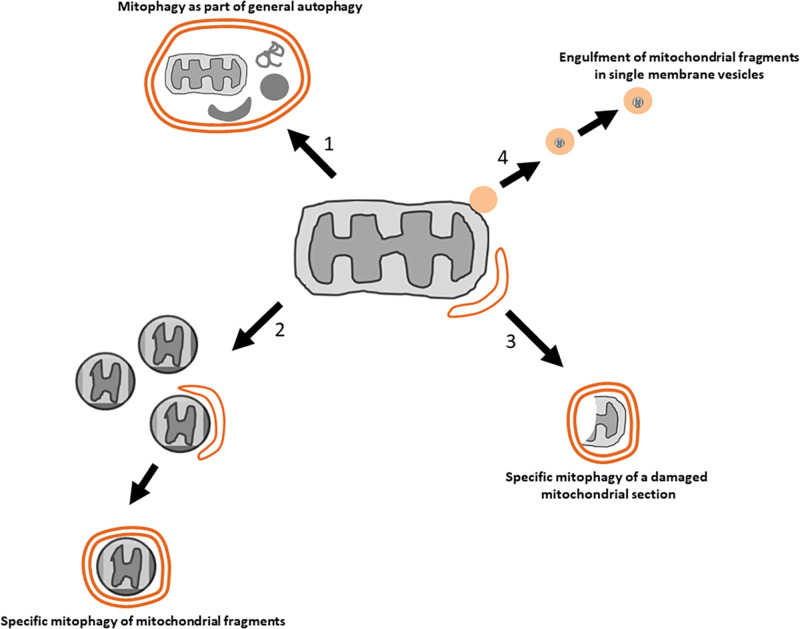
Pathways of mitochondrial degradation. As part of general, non-selective autophagy, mitochondria can be included in the cargo of autophagosomes together with other components (scheme 1). Mitochondria can also be fragmented and then eliminated after fragmentation, as part of a specific mitophagy process (scheme 2). Damaged mitochondrial regions can be eliminated by engulfment into autophagosomes assembled on the damaged site leaving the rest of the organelle intact (scheme 3). A distinct pathway of elimination of mitochondrial components involves the formation of mitochondrial derived vesicles that transport cargo to lysosomes for degradation (scheme 4).

The pathway of autophagy generates nutrients during periods of starvation and eliminates faulty cellular material as part of a quality control process. Morphological analysis of this pathway from the earliest studies revealed that mitochondria were frequently found engulfed in autophagosomes (Figure 1, scheme 1). For example, amongst the recognisable sequestered components of autophagosomes from rat liver following 3 h of autophagy induction, mitochondria constituted 25% of cargo [1]. [Parenthetically, fragments of endoplasmic reticulum topped the list at 31%.] It is still not clear how these mitochondria are targeted during starvation-induced or basal autophagy. Are they simply engulfed as part of the general cargo that is indiscriminately incorporated into forming autophagosomes, or is there some specificity/preference for those organelles that are damaged in some way? Answering these questions is extremely important given the critical role of healthy mitochondria in cell physiology [2,3].

In contrast, much is known about the pathway of mitochondria-specific autophagy (mitophagy) which I define here as the elimination of mitochondria following distinct mitochondrial injuries or developmental or metabolic alterations. Many aspects of mitophagy, from the various conditions that trigger it to its physiological functions in health and disease, have been recently reviewed [4,5] while early foundational studies have also been retrospectively discussed [6]. This brief review will be focussed on the earliest steps of the pathway concerning the mechanisms and requirements for initiating autophagosome formation on a fragmented mitochondrial target (Figure 1, schemes 2 and 3). In view of the fact that we understand the basic mechanism of non-selective autophagy and are beginning to reshape this knowledge towards the pathways of selective autophagy, two aspects of mitophagy will be covered:

- How does a machinery normally working in association with the endoplasmic reticulum (ER) to make an autophagosome, can also do so at a site distinct from the ER, such as on the surface of the targeted cargo?
- Does the machinery need to measure the targeted cargo, or, to put it another way, how does the machinery deal with cargo of multiple sizes?

Another pathway involving transfer of mitochondrial components to the lysosomes for degradation via mitochondrial-derived vesicles has been described with some overlap with mitophagy regarding the machineries involved [7,8] (Figure 1 scheme 4). However, this pathway does not generate double membrane autophagosomes and will not be considered further here.

## Early steps in non-selective autophagosome formation

**Figure 2.**
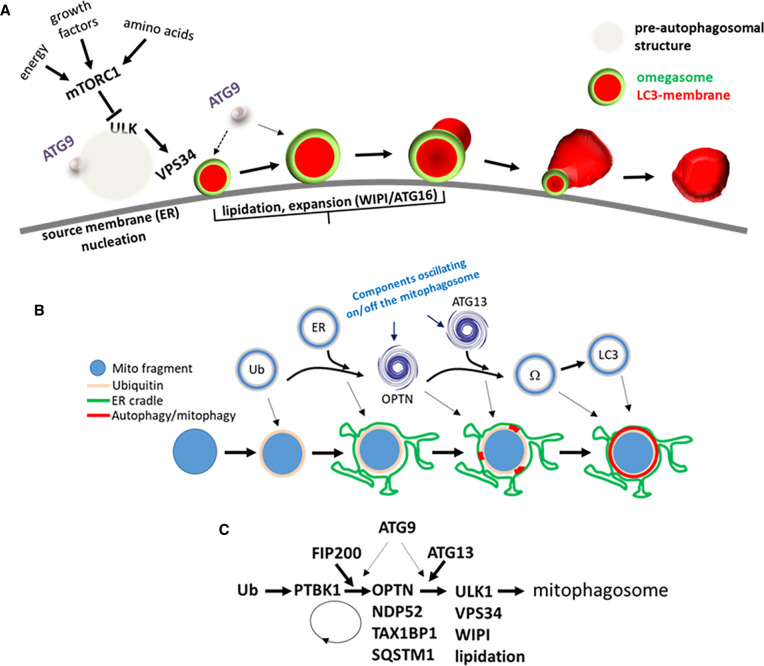
General autophagy pathway and its modification during ivermectin-induced mitophagy. (**A**) During autophagy, amino acids, growth factors and other signals stop activating mTOR which in turn leads to the activation of the ULK complex, the VPS34 complex and ATG9 vesicles on pre-autophagosomal regions connected to the ER and likely neighbouring phase separated assemblies (opaque circle). In the next step, VPS34 synthesises PI3P on ER-connected omegasomes which nucleate autophagosome formation by attracting the LC3 lipidation machinery. Eventually, autophagosomes separate from omegasomes and traffic to the lysosomes for degradation. (**B** and **C**) The specific ubiquitin-dependent engulfment of mitochondria during mitophagy depends on ubiquitination of mitochondrial fragments and then their association with ER strands. Engulfment is co-ordinated by mitophagy receptors (shown here for OPTN) and early autophagy proteins (shown here for ATG13) that oscillate on and off the forming mitophagosome, followed by omegasome formation (Ω) and the LC3 lipidation machinery. These sequential membrane rearrangements depend on a series of translocations of autophagy and mitophagy components to the targeted mitochondrial fragments as shown in **C**. Note the functional separation of FIP200 and ATG13, both components of the ULK complex. Elements of this figure have been modified from ref. [71].

Conditions of amino acid scarcity trigger the inactivation of the mammalian (mechanistic) target of rapamycin complex 1 (mTORC1) which leads to the activation of the ULK complex (composed of the kinases ULK1 or ULK2 and the adaptors FIP200, ATG13 and ATG101) and its subsequent translocation to regions associated with the ER that become nucleation sites for autophagosome formation [9–13] (Figure 2A, left side). These pre-autophagosomal sites are still under intense investigation. Combination of live imaging and FIB-SEM studies have shown them to be tubulovesicular elements formed by ULK complex assemblies and ATG9 vesicles surrounded by ER membranes [14]. Very recent data in both yeast and mammalian cells have suggested that another important element specifying pre-autophagosomal structure formation are biomolecular condensates resembling phase separated liquid droplets and containing a small group of early autophagy proteins such as the ATG1 complex in yeast (equivalent to the ULK1 complex in higher eukaryotes) and p62 (an adaptor protein involved in non-selective and selective autophagy) in mammalian cells [15–17]. How these condensates contribute to autophagosome formation is still a matter of speculation [18–20]. Two of the general properties of condensates, their enhancement of protein–protein interactions within the restricted milieux of the droplet and their enabling of protein-membrane interactions at the periphery of the droplet [21] may hold the key. Fujioka et al. [16] suggest that the condensates facilitate ATG1 complex function during autophagy whereas Agudo-Canalejo et al. show that autophagosomes form at the edges of liquid droplets composed of p62 and this topography aids membrane bending during the process of autophagosome closure [15]. There is another possibility worth contemplating. A forming autophagosome likely makes contact with a number of intracellular membranes [22] and this characteristic has made the origin of the autophagosomal membrane a question with multiple answers [23]. In this view, interactions between the early autophagosomal structure and various intracellular membranes — with a primary role for the ER - could take place at the periphery of the liquid droplet thus providing a spatial restriction and guidance for the formation of such contact sites. Of note, a similar membraneless organelle intertwined with the ER and allowing translation of specific mRNAs has recently been proposed [24].

The next stage of autophagosome formation requires the ULK-mediated activation of the VPS34 complex (composed of the type III PI-kinase VPS34 and the adaptor proteins VPS15, BECLIN1 and ATG14) which is responsible for phosphorylation of PI to generate PI3P at sites connected to the ER and termed omegasomes [25,26] (Figure 2A, middle). The function of omegasomes is three-fold. They serve as membrane platforms within which autophagosomes are formed [27], they help bring to the formation site the machinery that covalently attaches PE on the major autophagy protein LC3 (and the rest of the ATG8 family members) which coincides with the growth of the autophagosomal membrane [28], and, finally, they allow tethering of the lipid transfer protein ATG2 between omegasomes on the ER and pre-autophagosomal structures thus enabling essential supply of lipids to the forming autophagosomes [29–31].

The role of ATG9, the third essential element (together with the ULK and VPS34 complexes) in the early steps of autophagosome formation, remained elusive until recently. In yeast, a finite number of ATG9-containing vesicles are thought to nucleate the autophagosome formation site [32–34]. In mammalian cells, live imaging experiments have suggested that a small ATG9-containing vesicular structure makes contact with the ER immediately before the translocation there of the ULK complex [14] whereas numerous ATG9 vesicles are seen at later times to interact with the forming autophagosome [35]. The latter observation can be explained by the recent identification of ATG9 as a lipid flipping and transporting protein very likely involved in normalising the distribution of newly incorporated phospholipids between the membranes of the forming autophagosome [30,36]. It is less clear how the initial contact between ATG9 vesicles and the ER regulates subsequent ULK complex recruitment. One hypothesis is based on the recent observation that, in addition to its lipid scrambling activity, the structure of ATG9 as a trimer suggests that the protein is able to bend or tubulate lipids [37]. Given that omegasome formation and subsequent expansion appear to be most frequently seen at ER tubular extensions [25,38] it is possible that, early during autophagy induction, ATG9 protein is delivered to the ER where it tubulates regions of the ER on which the initiating machinery (ULK and VPS34 complexes) coalesces to nucleate autophagosome formation. This speculative idea is obviously in need of experimental examination.

## Formation of a mitophagosome: ‘eat-me' signals, adaptors and receptors

In analogy to non-selective autophagy as outlined above, early work has shown that the ULK complex and ATG9 vesicles independently target mitochondrial for degradation [39], whereas omegasomes are involved at later stages [40]. However, several other machineries are also involved in mitophagy, and a major challenge has been to understand how they co-ordinate in space and time with the canonical autophagy proteins in order to engulf the targeted structure [41,42] (Figure 2B,C). Selective autophagy must rely on a cargo-specific ‘eat me' signal to mark the cargo for engulfment, and on additional mechanisms to connect the targeted cargo to the autophagic machinery [43,44]. Signals for autophagic degradation are either ubiquitin-dependent or ubiquitin independent [42,45]. In the first case, ubiquitin molecules usually in phosphorylated form are attached to damaged mitochondria for autophagic targetting [46–48]. One of the best-understood pathways *in vitro* that mediates ubiquitination of damaged mitochondria and mitophagy is the PINK1/PARKIN pathway [49]. In the healthy state, the kinase PINK1 normally residing in the mitochondrial interior is exported to the cytoplasm and degraded. When mitochondria are depolarised due to chemical or biological injury, PINK1 is stabilised and phosphorylates ubiquitin bound at low levels to outer membrane mitochondrial proteins [46,50,51]. PINK1 also binds to the E3 ligase PARKIN and phosphorylates it, which in turn increases ubiquitination of mitochondrial proteins resulting in enrichment of ubiquitin molecules (‘eat me' signals) on mitochondria [52–54]. Strong overexpression of PARKIN is sufficient to eliminate all of the cellular mitochondria that have been depolarised with chemical uncoupler within a few hours [55].

In the case of ubiquitin-independent pathways, a number of mitophagy receptors are recruited to damaged mitochondria (or exposed on their surface) to mediate interaction with the autophagic machinery [2,56–58]. BNIP3 and BNIP3L (or NIX) are two such receptors which regulate mitophagy under hypoxic conditions with BNIP3L also being essential for mitochondrial clearance during erythrocyte maturation and somatic cell reprogramming [2,56]. Both BNIP3 and BNIP3L contain oligopeptide domains that interact with ATG8 family proteins (LIR domains) and this is the mechanism for autophagosome recruitment. Interestingly, BNIP3L preferentially interacts with GABARAP and GABARAPL1 of the ATG8 family whereas BNIP3 (especially upon phosphorylation) binds to LC3B and GABARAPL2 in preference to the other ATG8 proteins. It is not clear if these differences in binding affinity correspond to physiological conditions; in general, the functional rationale for the redundancy of the ATG8 family proteins is still unknown. Another receptor involved in mitophagy independently of a ubiquitin ‘eat me' signal is FUNDC1, a transmembrane protein of the outer mitochondrial membrane that has been implicated in homeostatic pathways of cardiac cells. Interaction of FUNDC1 with the autophagic machinery during hypoxic conditions involves both a LIR domain exposed on the cytosolic side as well as binding to the ULK1 kinase [2,56]. All of these interactions between receptors and ATG8 family members are regulated by phosphorylation cascades thus modulating receptor affinity for the forming autophagosomal membrane. It is less clear how the upstream autophagic machinery (including the ULK and VPS34 complexes as well as ATG9 vesicles) is also recruited to mitophagosomes formed during ubiquitin-independent mitophagy. Lessons learned from the ubiquitin-dependent pathways (see below) would suggest that these receptors must have the ability to interact with the upstream machinery; undoubtedly, future work will address this question.

For ubiquitin-dependent pathways, autophagy adaptors including p62/SQSTM1, NBR1, NDP52, TAX1BP1, and OPTN recognise the ubiquitin signal via specific domains and translocate to damaged mitochondria [43,59,60]. An important element of recognition of the autophagic machinery by the adaptors is the presence of LIR domains that allow binding to the ATG8 family [43,59,60]. Thus, by combining a domain that recognises ubiquitin with another that recognises the ATG8 proteins, adaptors bridge the space between cargo and autophagosomal membrane.

Although in principle the interaction between ATG8 family proteins and adaptors could suffice to bring the autophagic machinery to the targeted cargo, the situation is more complex because early autophagy proteins, including the ULK complex, ATG9 and the VPS34 complex effectors can also directly interact with the mitophagic recognition machinery. For example, the PI3P binding protein WIPI2 was shown to translocate to bacteria-directed autophagosomes following activation of TBK1, a kinase that activates by phosphorylation a number of mitophagy adaptors [61] whereas the ULK complex component FIP200 interacts directly with p62/SQSTM1 during selective autophagy [62]. Similarly, NDP52 was shown to interact with the ULK complex (especially the FIP200 protein) during selective autophagy of mitochondria or bacteria [63,64] and the receptor Bcl2-L-13 induces the translocation of the ULK complex during mitophagy [65]. Another mitophagy adaptor, OPTN, was shown to interact directly with ATG9 during the engulfment of mitochondria [66]. In view of the above, it is perhaps not surprising that engulfment of cargo during selective autophagy need not rely on the ATG8 family at all but can use instead the ATG4 proteins for autophagosomal membrane expansion [67].

Of particular interest is the observation that the p62/SQSTM1 adaptor as well as ubiquitin molecules condense on liquid droplets during the process of autophagosome formation [17,62]. This could provide an organising mechanism for adaptors and early autophagy proteins to nucleate selective autophagosome formation in analogy to non-selective autophagy.

The way that these adaptors and the autophagic machinery co-operate to target mitochondria in real time will be discussed in the next section. Here, it is worth noting that this efficient and rapid engulfment relies on two inter-related positive feedback loops [5,42,48]. One involves the ubiquitination signal that is amplified by phosphorylation as mentioned above. A small amount of ubiquitin is present on mitochondria even under basal conditions and these molecules are phosphorylated upon depolarisation and PINK1 activation. Phosphorylated ubiquitin further recruits PARKIN resulting in additional ubiquitin molecules attached to mitochondria which are also phosphorylated. This cycle of PINK-induced ubiquitin phosphorylation followed by PARKIN recruitment and additional ubiquitination rapidly coats the targeted mitochondria by ubiquitin at the initiation stage of mitophagy. A second positive feedback loop involves phosphorylation of the mitophagy adaptors by the kinase TBK1. This phosphorylation increases affinity of adaptors for ubiquitin and for ATG8 proteins, thus enhancing the interaction between adaptors and autophagosomal membranes. Importantly, phosphorylation by TBK1 also enhances the association of adaptors with TBK1 itself and with early autophagy proteins such as those of the ULK complex. Therefore, a positive feedback loop between PTBK1-mediated phosphorylation of adaptors followed by additional recruitment of TBK1 for subsequent adaptor phosphorylation would provide rapid nucleation of autophagic components in the vicinity of the targeted mitochondrion. These findings have been reported for the PINK1/PARKIN-dependent pathway but, given that other ubiquitin-dependent pathways rely on similar signals, it is likely that they will be widely applicable.

## Dynamics of mitophagy

During mitochondrial engulfment, several proteins and protein machineries must co-ordinate: the ubiquitin signal, the adaptors/receptors, and the early autophagy components including ATG9 vesicles, the ULK complex and the VPS34 complex (Figure 2B,C). Live imaging studies are beginning to explain how this complicated process is organised. During PINK1/PARKIN mitophagy caused by light illumination of a mitochondrially targetted photosensitizer, small pieces of damaged mitochondria detached from the main membrane in regions where ER strands contact mitochondria membranes [68]. These detached pieces were positive for omegasome markers and LC3 after they became positive for ubiquitin and PARKIN, indicating that the formation of ‘eat-me' signals precedes their recognition by the autophagic pathway [68]. During mitochondrial damage with the mitochondrial uncoupler carbonyl-cyanide m-chlorophenylhydrazone (CCCP), PARKIN was recruited first to the damaged (ubiquitinated) regions followed by OPTN, one of the essential mitophagy adaptors [69]. This was subsequently followed by omegasome formation and LC3 translocation [69]. Interestingly, in the absence of PARKIN, OPTN still translocated to damaged regions but transiently without leading to omegasome recruitment. In an expansion of this work, all three mitophagy adaptors OPTN, NDP52, and TAX1BP1 were shown to be recruited to damaged mitochondria with very similar kinetics and preceding the recruitment of the omegasome-localizing autophagic machinery [70]. Although all mitophagy adaptors were co-recruited to damaged sites, OPTN had a more essential function together with the TBK1 kinase which was also recruited early to these damaged mitochondrial sites [70]. Close inspection of the dynamics of OPTN and omegasome recruitment in the work by Wong and Holzbaur suggests that these two components do not coincide spatially on the damaged mitochondrial fragments but appear to mark distinct regions (see for example Figure 5C,D in ref. [69]). I will return to this point later.

**Figure 3.**
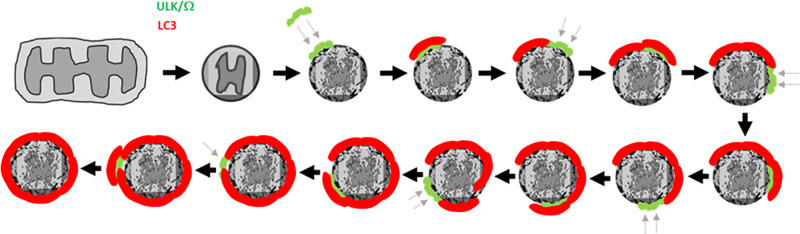
How a large target can be engulfed during selective autophagy. Mitochondrial fragments are targeted for autophagic engulfment by sequential translocations of ULK complex, enabling VPS34 mediated omegasome formation and generation of LC3-containing phagophores at distinct regions each time. The ULK and omegasome components are indicated by the green structures and the LC3-containing phagophores are labelled in red. Note that phagophore formation is piecemeal (small pieces are made each time) and is preceded by translocation of the ULK/omegasome structures at each step. Reiteration of this process until all surface of the mitochondrial target is covered and engulfed will produce oscillatory behaviour of the ULK complex as we observed during live imaging. How the small phagophores may be combined is unknown at this point. This figure taken from ref. [72].

All preceding live imaging work was done in cells overexpressing PARKIN. To move away from this protocol, we used ivermectin to damage mitochondria and then followed mitophagy within a few minutes of treatment [71] (Figure 2B,C). This compound causes fragmentation of mitochondria and a reduction in the oxygen consumption rate [71]. Because these fragmented mitochondria become ubiquitinated and engulfed by the autophagic machinery within 30 min of treatment, ivermectin provides a useful tool for studying the dynamics of mitophagy [71]. In our imaging studies, we followed several components including the ER, the ubiquitin signal, the ULK complex as well as adaptors, omegasomes and LC3. We discovered that in this mitophagy protocol a very early event is the cradling of ubiquitinated mitochondrial fragments within ER strands, and it is within those membranes that the rest of the mitophagy/autophagy machinery forms. Using both live imaging and morphological measurements of mouse embryonic fibroblasts deficient in various autophagy genes or treated with inhibitors of the early components, we found that FIP200 and TBK1 translocated early followed by the adaptors, the rest of the ULK complex and the omegasomes with their effectors [71]. We observed surprising dynamics of the ULK complex (as exemplified by ATG13) during mitophagy. Instead of a single translocation to the forming mitophagosome, the ATG13-containing puncta translocated on and off several times until completion of the engulfment by LC3-containing membranes. The basis of the oscillatory behaviour of ATG3 is that each mitochondrial fragment is covered sequentially by several early autophagosomal structures (phagophores) starting at different times and locations which are ‘stitched' together to form the complete mitophagosome [72] (Figure 3). I will expand on this point below.

## Large autophagy targets are covered by multiple phagophores which are then combined together

**Figure 4.**
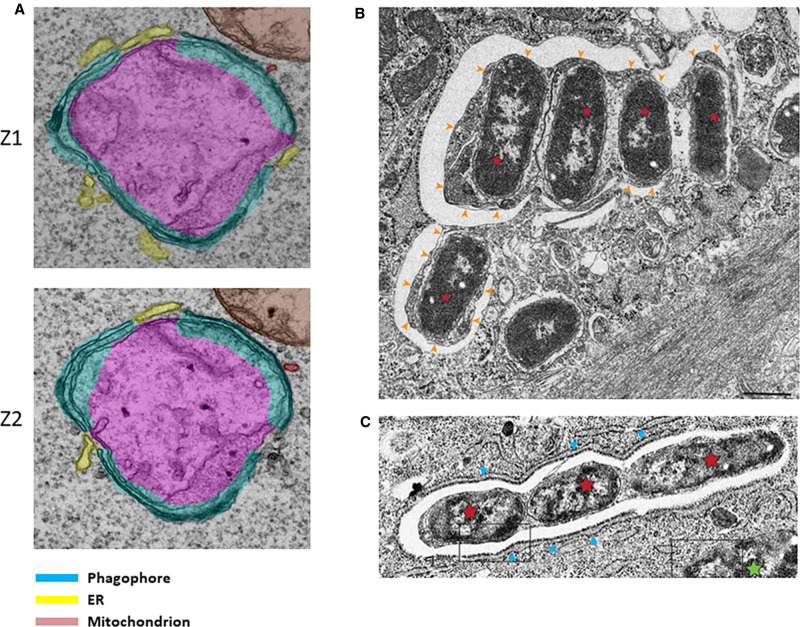
Characteristics of mitochondrial and bacterial engulfment during autophagy. (**Α**) Two sequential slices from a tomographic reconstruction of ivermectin-induced mitophagy are shown. Note that three phagophores are assembling on this mitochondrial fragment, and that the ER appears to occupy areas not covered by the phagophores. Colour scheme refers to this panel only. (**B** and **C**) During autophagy of *Salmonella* it is common to see multiple phagophores forming around bacterial clusters (**B**, bacteria are labelled with red asterisks and phagophores with yellow arrows) whereas the ER is frequently seen outlining the autophagosomal structures (**C**, ER labelled with blue arrows and bacteria with red asterisks). Mitophagy images shown in panel **A** are modified from ref. [71] whereas *Salmonella* images in **B** and **C** are modified from ref. [74].

What is the explanation for the dynamic behaviour first observed for OPTN by Wong and Holzbaur [69] and reproduced in our live imaging studies for ATG13 during engulfment [71]? To address this question it is important to remember that the autophagic machinery can engulf cellular structures as small as a few nm in diameter (for example glycogen or protein aggregates) and as large as bacteria and mitochondria measuring a few μm in diameter [73]. When we synchronised the oscillatory dynamics of ATG13 during several mitochondrial engulfment events we noted that the number of oscillations varied between events, although the overall spacing and amplitudes were not very different [72]. Comparing the number of oscillations to diameter of the mitochondrion being engulfed, we obtained an almost linear relationship which indicated that the size of the targeted structure correlated with how many times the ATG13 particles translocated to it. But why this oscillatory behaviour? We hypothesised that such large structures require multiple rounds of engulfment, with each round resulting in a small phagophore covering only a portion of the targeted structure. In this hypothesis, each oscillation represents the nucleation of the ATG13 protein (as a surrogate for the entire ULK complex) to make a phagophore at a small region of the mitochondrion, a process which then repeats itself until the entire structure is covered (Figure 3). We modelled this, adding a parameter for increasing delays of ATG13 finding an empty region as the engulfment proceeds to completion, and obtained a graph very similar to the observed oscillatory data [72], suggesting that such a piecemeal engulfment process can give rise to the oscillatory behaviour of ATG13 (and presumably OPTN). There is experimental evidence for the idea that engulfment of large structures proceeds in a piecemeal fashion. Our work on mitophagy examined by live imaging and electron tomography revealed that large mitochondrial fragments are surrounded by multiple phagophores before a continuous autophagosome is formed [71] (Figure 4A). Similar structures composed of multiple phagophores and surrounding a single ruptured *Salmonella* bacterium or multiple bacteria clustered together have also been observed during bacterial autophagy [74,75] (Figure 4B,C). It therefore may be a common property of autophagic engulfment — especially for large targets — that the process advances in a piecemeal fashion making small phagophores before the discreet structures are fused together (Figure 3). In the future, it will be important to determine the mechanism of fusion of the partially curved planar membrane sheets and the co-ordination of this step during engulfment.

## Role of the ER

Early work on mitophagy mediated by PINK1/PARKIN suggested that phagophore membranes were associated with the rough ER [76] on the outside of the autophagosomal membrane and not threading in and out as is the case for starvation-induced, non-selective autophagy [77,78]. Other live imaging work also suggested that mitophagy takes place at the junction points between ER and mitochondria [68] whereas even ATG8-independent autophagosome formation during mitophagy appears to depend on ATG4 family proteins mediating contact between mitochondrial targets and ER [67]. As mentioned above, we have also observed in our work on ivermectin-induced mitophagy that the ER surrounded targeted mitochondria as early as the ubiquitination step and maintained this association even as the forming mitophagosome travelled around the cell before its completion [71]. Live imaging coupled with electron tomography to capture mitophagic structures at the highest resolution showed that autophagic membranes appeared to extend from ER strands and surrounded the targeted mitochondrial fragments [71] (Figure 4A). In other super-resolution microscopy work, we captured many examples of ER surrounding the targeted mitochondria during mitophagy [71]. In those images, the ER appeared to cradle both the ubiquitin signal and the downstream early mitophagy and autophagy components [71]. The intimate involvement of the ER during mitophagy can be rationalised because the machinery that generates autophagosomes during starvation-induced autophagy (i.e. the ULK complex, ATG9 vesicles and the VPS34 complex with its effectors) readily uses the ER as a cradle for autophagosome formation and as a membrane source [23]. Therefore, if mitochondrial fragments destined for autophagic engulfment are surrounded by ER, the process of nucleation and expansion of autophagosomal membranes can be straightforward. Of note, during *Salmonella* autophagy, similar close associations between the ER and the forming autophagosomes (either single or multiple structures assembling together) were seen by EM [74] indicating that the ER may be involved in several different types of selective autophagy pathways (Figure 4C). An ER cradle during selective autophagy may also be of benefit when the pathway proceeds in a piecemeal fashion creating small phagophores that are fused together (as discussed above). In our live imaging experiments, we frequently observed mitochondrial fragments travelling long distances in the cell as they were being engulfed by the autophagic machinery [71], and, in general, mitophagy rarely proceeded on immobile structures. Interestingly, the ER cradle also moved along the targeted mitochondria during this process. It is therefore possible to imagine that a re-iterative process (the piecemeal engulfment) targeting a moving object may be better completed if it takes place on a restricted platform such as the one provided by an ER cradle. An additional aspect of this that is worth exploring further is whether the condensates/liquid droplets that may nucleate early autophagic structures are also dynamically restricted by the ER strands as they form.

Are there no cases where autophagosomes form directly on the targeted membrane? One example may be during mitophagy induced by hypoxia or iron chelation [79]. In this experimental setting, and for at least some cell lines, mitochondria do not fragment before mitophagy (as is the case for most other mitophagy induction protocols), but, instead, a small damaged region attracts autophagic machinery which assembles there and pinches off the damaged region leaving the rest of the mitochondrion intact [79] (Figure 1 scheme 3). This is a very interesting mechanism likely to be relevant in physiological settings where damage is localised and it would be wasteful to eliminate whole mitochondria because of it. However, how the autophagic machinery targets these regions and the details of the pinching off step are questions to be addressed in the future. It is likely that this pathway may utilise lipids being supplied by the mitochondrion itself for the formation of a small autophagosome.

## Conclusion and current unknowns

The dynamics of mitophagy initiation appear to follow similar sequential steps as the orderly translocations of the autophagy machinery during the formation of non-selective autophagosomes. The major differences between the two pathways is the ubiquitination step and the engagement of the mitophagy adaptors. However, both of these additional steps also exhibit temporal dynamics, with ubiquitination at the beginning of the process and the translocation of the mitophagy adaptors at a later step. This translocation is based on the recognition of the ubiquitinated cargo and it enables the subsequent engagement of the ULK complex proteins. One interesting difference may be that FIP200 works before, or concomitantly with, the mitophagy adaptors, but before the rest of the autophagy machinery.

Our understanding of mitophagy in organismal settings lags far behind our understanding in tissue culture cells following experimentally induced damage. This is currently being addressed with various probes that report on mitophagy in intact cells and tissues [80–82] with early data indicating significant differences in the extent of this process between tissues and developmental stage. At the same time, the actual mechanism by which mitochondrial membranes segregate into normal and damaged entities before damaged ones are eliminated is also being addressed and it appears to depend on the DRP1 protein and contact sites either with the ER or with lysosomes [83]. These types of approaches coupled with the ever-increasing resolving power of whole tissue microscopy are likely to allow us a much better view of the process of mitophagy in the truly relevant physiological setting.

## Perspectives

- Healthy mitochondria are important constituents of healthy cells, and mitophagy is a quality control process that eliminates damaged mitochondria.
- The pathway of mitophagy is beginning to be understood in great mechanistic detail because of the deep understanding of general autophagy and of the components that target mitochondria for elimination.
- Our understanding of mitophagy is less detailed at the tissue and organismal level; new mitophagy indicators that work in whole animals will provide such knowledge in the future.

## Abbreviations

CCCP: carbonyl-cyanide m-chlorophenylhydrazone
ER: endoplasmic reticulum
mTORC1: mammalian (mechanistic) target of rapamycin complex 1
